# *Helicobacter pylori* γ-glutamyl transferase contributes to colonization and differential recruitment of T cells during persistence

**DOI:** 10.1038/s41598-017-14028-1

**Published:** 2017-10-20

**Authors:** Stefanie Wüstner, Florian Anderl, Andreas Wanisch, Corinna Sachs, Katja Steiger, Andreas Nerlich, Michael Vieth, Raquel Mejías-Luque, Markus Gerhard

**Affiliations:** 1 Institut für medizinische Mikrobiologie, Immunologie und Hygiene, Technische Universität München, 81675 Munich, Germany; 2 Institute of Pathology, Technische Universität München, 81675 Munich, Germany; 3 Institut für Pathologie, Klinikum München-Bogenhausen, 81925 Munich, Germany; 4 Institut für Pathologie, Klinikum Bayreuth, 95445 Bayreuth, Germany; 5 German Centre for Infection Research (DZIF), partner site Munich, Munich, Germany

## Abstract

*Helicobacter pylori* γ-glutamyl transferase (gGT) is a key bacterial virulence factor that is not only important for bacterial gastric colonization but also related to the development of gastric pathology. Despite accumulating evidence for pathogenic and immunologic functions of *H*. *pylori* gGT, it is still unclear how it supports gastric colonization and how its specific effects on the host’s innate and adaptive immune responses contribute to colonization and pathology. We have compared mice showing similar bacterial load after infection with gGT-proficient or gGT-deficient *H*. *pylori* to analyse the specific role of the enzyme during infection. Our data indicate that *H*. *pylori* gGT supports initial colonization. Nevertheless, bacteria lacking gGT can still colonize and persist. We observed that the presence of gGT during infection favoured a proinflammatory innate and adaptive immune response. Notably, *H*. *pylori* gGT activity was linked to increased levels of IFNγ, which were attributed to a differential recruitment of CD8^+^ T cells to the stomach. Our data support an essential role for *H*. *pylori* gGT in gastric colonization and further suggest that gGT favours infiltration of CD8^+^ cells to the gastric mucosa, which might play an important and yet overlooked role in the pathogenesis of *H*. *pylori*.

## Introduction

Over 50% of the world’s population persistently carries *Helicobacter pylori*, which is the main cause of gastric inflammation and more severe gastroduodenal diseases, such as gastric and duodenal ulcers, gastric cancer, or lymphoma^1^. Host, environmental and bacterial factors determine the inflammatory response towards the bacterium and thus the outcome of the disease.

*H*. *pylori* infection is associated with an increased influx of innate and adaptive immune cells into the stomach and production of proinflammatory cytokines^2,3^. During natural and experimental *H*. *pylori* infection in humans, gastric T lymphocytes are increased^4,5^ and T cell activation marker-positive (CD25^+^ and CD69^+^) cells are present at higher numbers compared to uninfected individuals^6^. Even though *H*. *pylori* is an extracellular pathogen, which are generally controlled by a Th2-driven antibody response, CD4^+^ T helper (Th) cells infiltrating the stomach rather display a Th1 and Th17 phenotype^7–9]^. Th cells produce a distinct set of cytokines. While IL-17 is the hallmark of a Th17 response, IFNγ is often used as a surrogate marker for a Th1 response. Studies with IFNγ-deficient mice demonstrated that IFNγ plays a major role in *H*. *pylori*-associated gastritis^10,11^. In addition, a regulatory T cell (CD3^+^/CD4^+^/FoxP3^+^) response is induced by *H*. *pylori* infection that is characterized by expression of IL-10 and TGFβ^3,12,13^.

Several virulence factors contribute to severity of *H*. *pylori*-associated pathology. Strong gastric inflammation and severe clinical manifestations in infected patients are, in part, attributed to the presence of cytotoxin-associated gene A (CagA) and vacuolating toxin A (VacA)^14,15^. These factors are directly - via a type 4 secretion system for CagA - or indirectly – by secretion and outer membrane vesicles for VacA - delivered to host cells and cause detrimental effects mainly on gastric epithelial cells^16,17^.

More recently, *H*. *pylori* γ-glutamyl transferase (gGT) has emerged as virulence factor that contributes to peptic ulcer disease and gastric cancer^18,19^. All *H*. *pylori* strains express gGT, stressing the fundamental function of this virulence factor. Thus, *H*. *pylori*-dependent glutaminase activity mediated by gGT, which results in the deprivation of glutamine and release of glutamate and ammonia into the periplasm as well as the surrounding environment, plays an important role in bacterial metabolism. For example, gGT-dependent production of ammonia has been shown to contribute to acid resistance of *H*. *pylori* ^20^. In addition, glutamate liberated by gGT can be taken up by *H*. *pylori* and is introduced into nitrogen and carbon metabolism^21^. Notably, gGT-dependent glutaminase activity not only contributes to bacterial metabolism, but also affects host’s epithelial and immune cells. Hence, glutamine deprivation, caused by *H*. *pylori* gGT activity, induces secretion of proinflammatory IL-8 in gastric epithelial cells^19^ and compromises T cell effector function^22^. Furthermore, *H*. *pylori* gGT activity drives dendritic cells towards a more tolerogenic phenotype favouring a regulatory T cell response, which might have consequences for immunopathology and persistence of the bacterium^23,24^.

Despite the fact that *H*. *pylori* gGT can directly affect host cells and at the same time is important for bacterial metabolism, the overall effect of this virulence factor during infection is not well understood. In this study, we have analysed the effect of gGT activity at different stages of infection in order to discriminate between the effects of the enzyme on the bacteria and the effects on host epithelial and immune cells. Moreover, we conducted a comprehensive analysis of the host’s immune response towards *H*. *pylori* infection in the absence and in the presence of gGT. Our results indicate that *H*. *pylori* gGT has a crucial role in gastric colonization and suggest that gGT enhances gastric inflammation and favours infiltration of CD8^+^ T cells to the gastric mucosa.

## Results

### *H*. *pylori* gGT activity contributes to the establishment of gastric colonization

**Figure 1:**
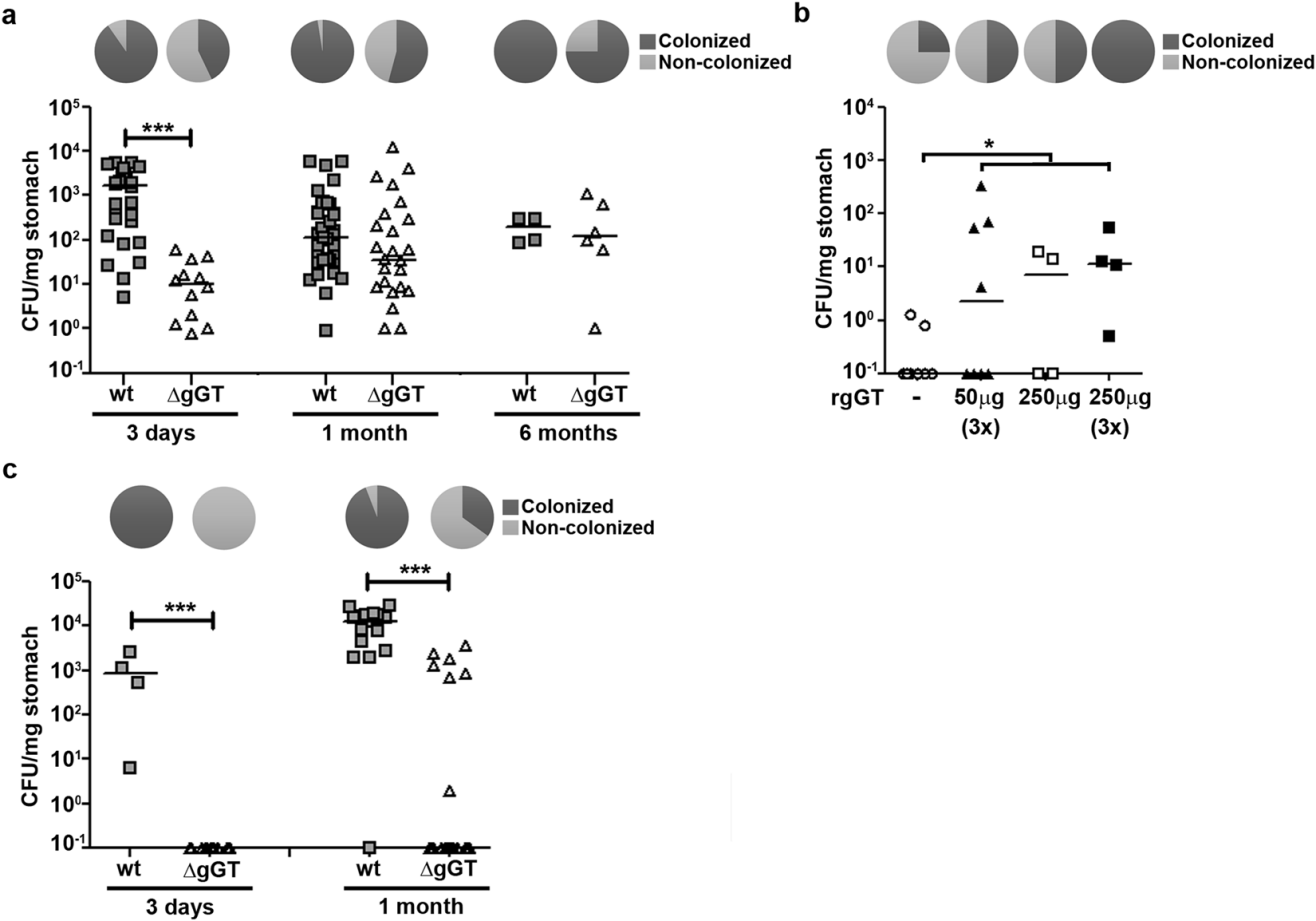
*H*. *pylori* gGT supports initial colonization. (**a**) Mice were inoculated with *H*. *pylori* PMSS1 (2 × 10^9^ CFU) wt or ΔgGT and sacrificed after three days, one month, or six months. Colonization level was determined by plating of serial dilutions. Mice from which bacteria could be grown at the time of analysis are shown. (**b**) C57Bl/6 mice were infected with *H*. *pylori* ΔgGT. 50 or 250 µg or recombinant *H*. *pylori* gGT were administered at the time of infection (1x) and on two consecutive days when indicated (3x). Data derived from one or two inoculations with four mice per group are shown. (**c**) Rag^−/−^ mice were infected with *H*. *pylori* wt or ΔgGT for three days and one month. Colonization levels of mice are shown. Mann-Whitney U test. *p ≤ 0.05, ***p ≤ 0.001. Horizontal bars indicate medians. Pie graphs indicate % of colonized mice.

It has been suggested that *H*. *pylori* gGT is essential for colonization but it has also been argued that it is required for persistence^24–26^. To define the role of *H*. *pylori* gGT during different stages of murine infection, C57Bl/6 mice were infected with *H*. *pylori* PMSS1 wt (wild-type, gGT-positive) or ΔgGT (gGT-negative).Gastric colonization was analysed after three days, one and six months (Fig. 1a). The overall rates of *H*. *pylori* infection were significantly higher in gGT-positive compared to gGT-negative strains (94.7% [71/75] vs. 52.4% [44/84]; p < 0.0001). At day three, gGT-negative *H*. *pylori* failed to establish an infection in 57.1% of inoculated mice; in contrast the gGT-positive *H*. *pylori* colonized more than 90% (Table 1). At one and six months post infection, 54.2% and 75% of mice harboured gGT-deficient *H*. *pylori*, while wt bacteria were detected in all but one mice infected with *H*. *pylori* wt. To confirm that the bacteria found in the stomachs of *H*. *pylori* ΔgGT-infected mice were still gGT-deficient, isolates were plated on agar plates containing or lacking kanamycin (Supplementary Fig. S1a). No significant differences in colony count were found in the presence or in the absence of kanamycin, indicating that the resistance cassette interrupting the *gGT* gene was still present.

**Table 1:** Colonization of C57Bl/6 mice infected with gGT-positive (wt) and gGT-negative (ΔgGT) *H*. *pylori*.

*H*. *pylori* strain^a^	3 days		1 month		6 months	
	wt	ΔgGT	wt	ΔgGT	wt	ΔgGT
number of independent inoculations	6	6	8	8	1	1
% noCFU (non-colonized/all mice)	9.7% (3/31)	57.1% (16/28)***	2.5% (1/40)	45.8% (22/48)***	0% (0/4)	25% (2/8)
CFU/mg stomach (all mice)^b^	661 (78.5–2947)	0 (0–7.9)^###^	107.5 (33.6–418)	1 (0–52.5)^###^	200 (89.5–304)	77 (0.3–511)
CFU/mg stomach (colonized mice)^b^	1693 (154–3134)	10.4 (1.5–31)^###^	111 (33.7–426)	35 (7.2–314)	200 (89.5–304)	122 (44.5–746)
% colonization rate per inoculation (range)	91.7% (75–100%)	50% (0–56.25%)	100% (100–100%)	50% (40–84.4%)	100%	75%

^a^C57Bl/6 mice were infected with 2 × 10^9^ *H*. *pylori* PMSS1 and analysed at different times post infection.

^b^Values are medians with interquartile range in parentheses.

^***^p ≤ 0.001, significant difference compared to *H*. *pylori* wt-infected mice according to Chi-squared test.

^###^p ≤ 0.001, significant difference compared to *H*. *pylori* wt-infected mice according to Mann-Whitney U test.

When only mice that were colonized at the time of analysis were considered, the median CFU per mg stomach was still significantly lower (164-fold) in mice colonized with gGT-negative compared to gGT-positive *H*. *pylori* at day three post inoculation (Fig. 1a and Table 1). At one month, this trend was still observable (3.2-fold), although it was not statistically significant. No differences in bacterial load were detected in mice colonized with *H*. *pylori* wt or ΔgGT for six months.

These results suggest that *H*. *pylori* gGT supports initial colonization. Once colonization is established, the CFUs observed in the stomach of *H*. *pylori* ΔgGT-infected mice increase to a similar level as observed in mice infected with the wt strain.

To confirm the supporting role of gGT for initial colonization and exclude a possible effect of the host’s immune response that would preferentially clear gGT-deficient bacteria, we performed histological analysis of the gastric mucosa after three days post-infection. Infected mice did not present innate or adaptive immune cell infiltration at this early stage of infection (Supplementary Fig. S1b,c). This confirms that the presence of gGT supports colonization itself and might represent a metabolic advantage for the bacteria. We further substantiated this hypothesis by externally supplementing recombinant gGT protein during infection. Notably, recombinant gGT was able to complement the gGT-deficient strain to some extent and allowed infection of a higher percentage of mice (Fig. 1b). Increasing the dose (50 or 250 µg) and frequency of application (one or three doses) of the recombinant enzyme increased infection rate with *H*. *pylori* ΔgGT.

Since *H*. *pylori* is secreted into the periplasm and also found in the extracellular space^26,27^, we postulated that gGT produced by wt bacteria might support colonization of *H*. *pylori* ΔgGT. Mice were inoculated with either *H*. *pylori* wt, *H*. *pylori* ΔgGT, or a combination of both at a 1:1 ratio. However, the proportion of mice colonized with *H*. *pylori* ΔgGT was even lower after coinfection compared to inoculation of the gGT-deficient strain alone (0% [0/17] vs. 42.9% [9/21]; p < 0.0001), while colonization level and rate of *H*. *pylori* wt was unaffected by coinfection (Supplementary Fig. S1d). Also, an indirect effect mediated by tolerization of dendritic cells by gGT released from wild type bacteria does not seem to facilitate colonization with *H*. *pylori* ΔgGT. Thus, these findings suggest that lack of gGT represents an important metabolic disadvantage for *H*. *pylori* and thus bacteria deficient for gGT are outcompeted by the wt.

Moreover, we studied the colonization capacity of wt- and gGT-deficient *H*. *pylori* in the absence of adaptive immune responses. Thus, Rag^−/−^ mice (lacking B and T cells) were infected with *H*. *pylori* wt or ΔgGT for three days or one month. After three days, no colony forming units were detected in the stomach of Rag^−/−^ mice infected with gGT-deficient *H*. *pylori* (Table 2 and Fig. 1c). After one month, *H*. *pylori* ΔgGT failed to establish an infection in more than 50% of mice, as it was observed in wt mice. This indicates that the same initial hurdle constrains colonization in absence of gGT activity in Rag^−/−^ mice as in wt mice. However, once the infection was established, gGT-negative *H*. *pylori* colonized Rag^−/−^ mice at a significantly lower level compared to gGT-positive bacteria (Table 2). These results indicate that the equalization of CFU observed in the long term infection of wt mice in the absence of gGT depends on the host’s immune response.

**Table 2:** *H*. *pylori* wt and ΔgGT colonization of immunodeficient Rag^−/−^ mice.

*H*. *pylori* strain^a^	3 days		1 month	
	wt	ΔgGT	wt	ΔgGT
number of independent inoculations	1	3	4	4
% noCFU (non-colonized/all mice)	0% (0/4)	100% (12/12)	5.9% (1/17)	65% (13/20)
CFU/mg stomach (all mice)^b^	840 (139.5–2202)	0 0^***^	13158 (3629–18093)	0 (0–816)^***^
% colonization rate per independent inoculation (range)	100% (100%)	0% (0)	100% (85–100)	38.3% (4.2–71–3)

^a^Mice were infected with 2 × 10^9^ *H*. *pylori* PMSS1 and analysed at 3 days or 1 month post infection.

^b^Values are medians with interquartile range in parentheses.

^***^p ≤ 0.001, significant difference compared to *H*. *pylori* wt-infected mice according to Mann-Whitney U test.

A substantial proportion of gGT-deficient bacteria were able to establish an infection (Fig. 1a). This implies that *H*. *pylori* might be able to adapt to and compensate for absence of gGT activity. Therefore, the colonization capacity of *H*. *pylori* ΔgGT re-isolated from stomachs of mice that were colonized for different times (10 days and 1 month) and at different levels (high [558 CFU/mg] and low [22 CFU/mg]) was tested. C57Bl/6 mice were inoculated either with the *H*. *pylori* ΔgGT parental strain or with bacteria that had been isolated from *H*. *pylori* ΔgGT-colonized mice. Presence of kanamycin and absence of gGT activity in *H*. *pylori* ΔgGT isolates was confirmed by PCR and a gGT activity assay (Supplementary Fig. S1e,f). Interestingly, all mice were colonized at one-month post infection with the gGT-deficient isolates tested, while the gGT-negative parental strain colonized only three of four mice (Supplementary Fig. S1g). In addition, the bacterial burden was less variable within the groups of mice infected with the isolates compared to mice infected with the parental strain. This indicated that *H*. *pylori* was indeed able to adapt to a lack of gGT activity with respect to colonization capacity.

### *H*. *pylori* gGT favours a proinflammatory innate immune response

We observed that *H*. *pylori* wt and ΔgGT established comparable levels of colonization at one month post infection (Fig. 1a). This allowed us to investigate gGT-dependent differences in the host’s immune response, since changes could be directly attributed to gGT activity and not to differences in bacterial burden.

**Figure 2:**
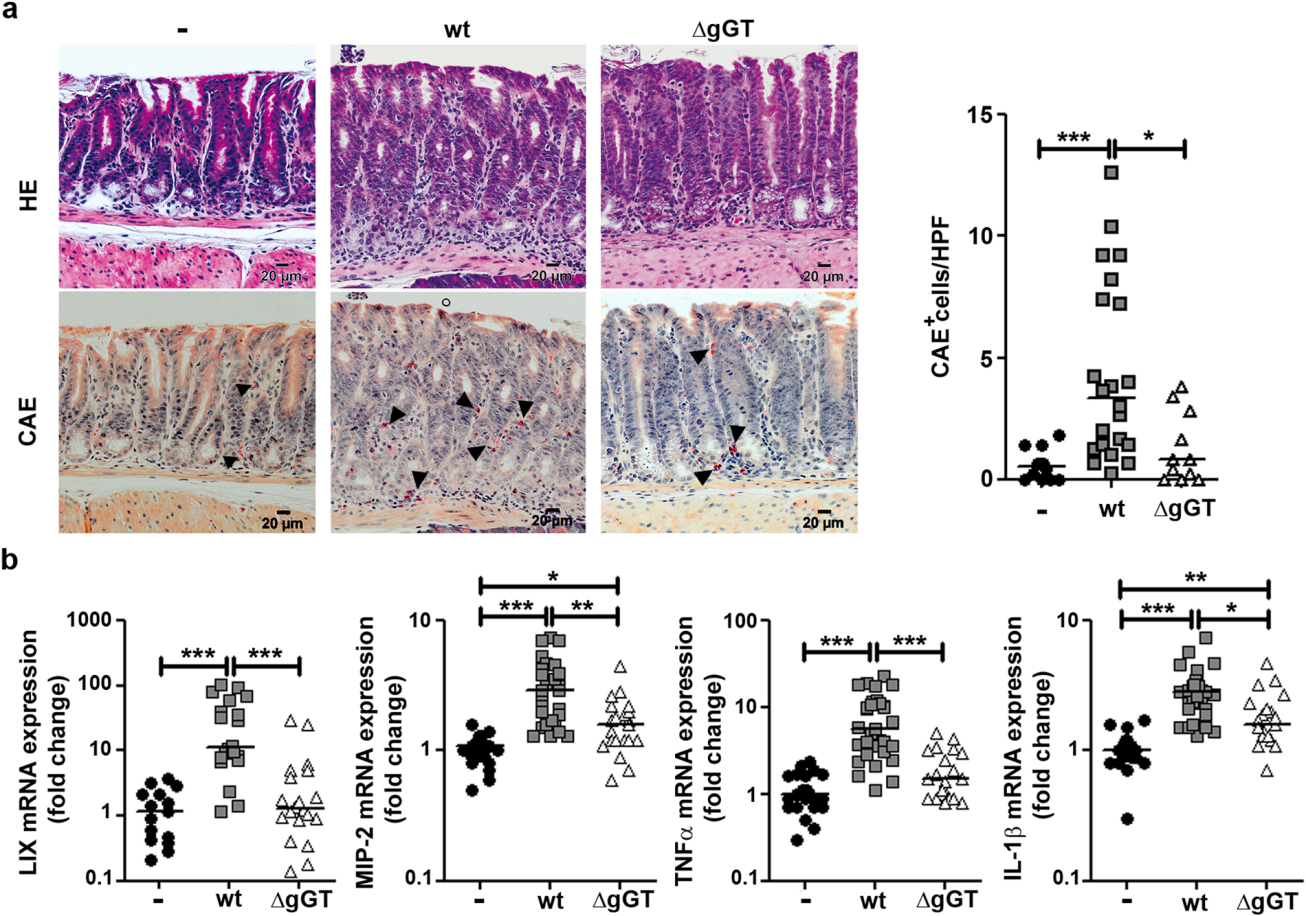
*H*. *pylori* gGT activity induces a strong innate immune response. C57Bl/6 mice were infected for one month. (**a**) Haematoxylin-eosin (HE) and chloroacetate esterase (CAE) staining of murine stomach sections. Images from representative mice of each group are shown. Number of CAE^+^ cells per high power field (HPF) was determined. Data are derived from three independent experiments. (**b**) mRNA expression levels of chemokines (LIX, MIP-2) and cytokines (TNFα, IL-1β) associated with the innate immune response determined by quantitative PCR. Results from four to six independent experiments are included. Kruskal-Wallis test followed by Dunn’s test for multiple comparisons. *p ≤ 0.05, **p ≤ 0.01, ***p ≤ 0.001. Horizontal bars indicate medians.

We first focused on innate immune responses and analysed infiltration of neutrophils into the stomach by chloroacetate esterase (CAE) staining of gastric tissue samples. *H*. *pylori* wt infection induced neutrophil recruitment to the stomach (Fig. 2a). Notably, presence of neutrophils was barely detected in the stomach of mice infected with *H*. *pylori* ΔgGT. After six months, no differences in gastric neutrophil infiltration were observed between mice infected with *H*. *pylori* wt and ΔgGT (Supplementary Fig. S2a,b).

Next, chemokines and cytokines expressed by innate immune cells and antigen-presenting cells including epithelial cells were assessed by quantitative PCR. In humans, CXCL5, also named epithelial-derived neutrophil-activating peptide 78 (ENA-78) since is mainly expressed by epithelial cells, is among the most strongly upregulated genes in human gastric mucosa upon infection with *H*. *pylori* ^28,29^. Interestingly, expression of the murine CXCL5 homologue, LIX, was strongly upregulated (11.3-fold) in mice colonized with *H*. *pylori* wt but not with *H*. *pylori* ΔgGT (Fig. 2b), despite of a similar bacterial burden. Another factor involved in neutrophil recruitment is IL-8. It is mainly produced by gastric epithelial cells upon infection with *H*. *pylori* ^30,31^. Thus, we analysed the expression of the murine IL-8 homologue, MIP-2. MIP-2 expression was higher in stomachs of mice infected with *H*. *pylori* wt compared to mice infected with *H*. *pylori* ΔgGT (2.9- vs. 1.6-fold) (Fig. 2b). We also analysed the expression of other cytokines involved in the innate immune response towards *H*. *pylori* such as TNFα and IL-1β. TNFα expression was induced 5.7-fold by colonization with *H*. *pylori* wt, while it was not induced by *H*. *pylori* ΔgGT (Fig. 2b). Thus, its expression was strongly gGT-dependent. Expression of IL-1β, mainly produced by monocytes and macrophages, was also significantly induced (2.8-fold) in mice colonized with *H*. *pylori* wt but at a lower level by colonization with *H*. *pylori* ΔgGT (Fig. 2b). Together these results indicate that the presence of gGT shapes innate immune responses against *H*. *pylori* towards a more proinflammatory phenotype.

### Absence of gGT activity results in similar T helper cell responses, yet leads to reduction of *H*. *pylori*-induced IFNγ expression

**Figure 3:**
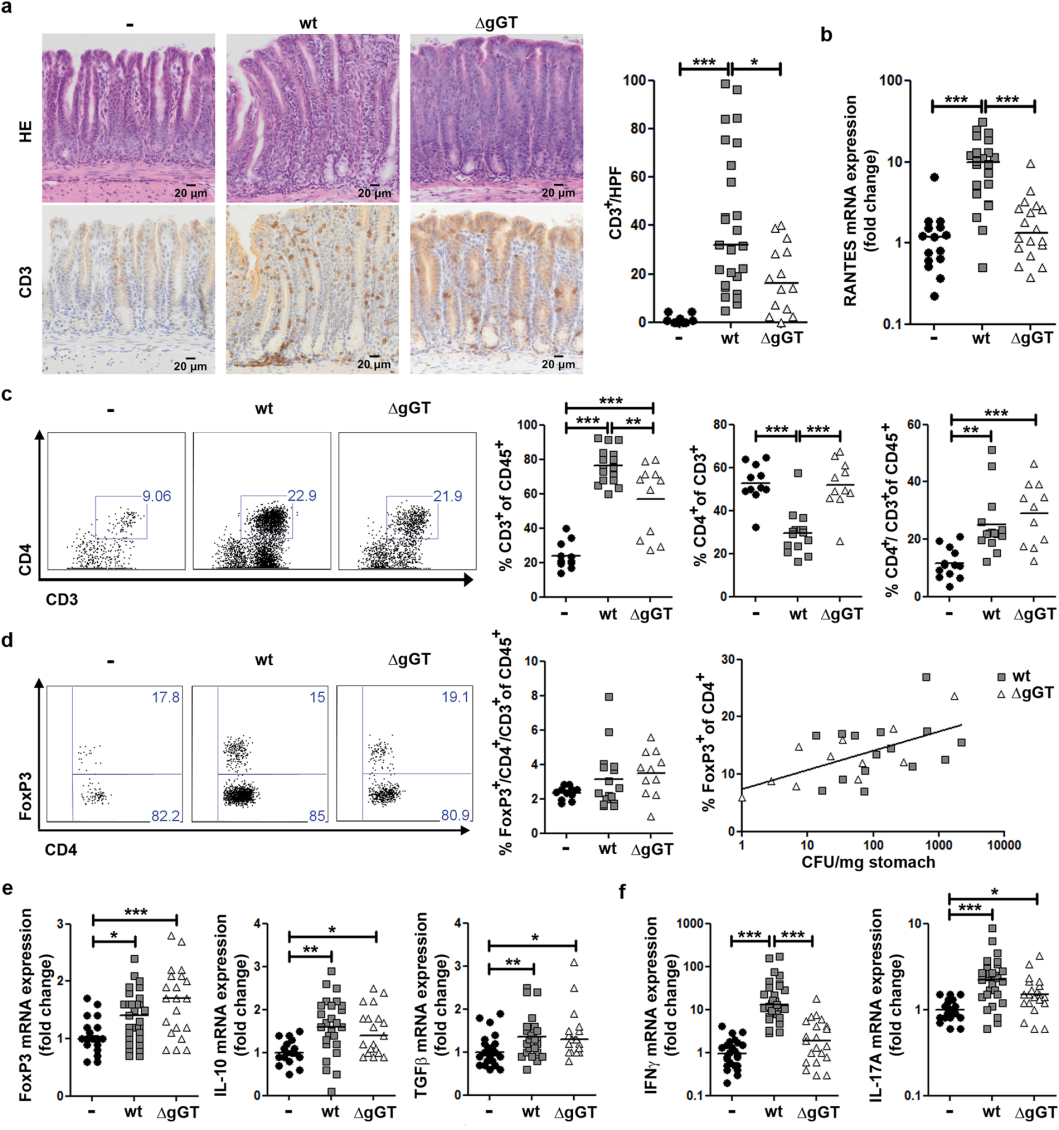
T cell response in mice infected with *H*. *pylori* wt or ΔgGT. Stomachs of mice were analysed one month post infection. (**a**) Representative images of Haematoxylin-eosin stain (HE) and CD3 expression in the stomach mucosa of mice from four independent experiments. Number of CD3^+^ cells per high power field (HPF) is shown. (**b**) mRNA levels of RANTES determined by quantitative PCR. Data depicted are derived from four independent experiments with four to eight mice per group. (**c**) Flow cytometry analysis of CD4^+^ T cell population in the gastric mucosa of mice. An exemplary dot plot of each group is depicted. (**d**) FoxP3-expressing CD4^+^ T cells. A representative dot plot of FoxP3 expression in CD4^+^ T cells is displayed. Proportion of FoxP3-expressing CD4^+^ T cells in the CD45^+^ leukocyte population and a positive non-linear correlation between the FoxP3-expressing CD4^+^ population and bacterial burden are shown (Spearman’s r = 0,7, p = 0.0204). (**e**) and (**f**) mRNA levels of cytokines associated with a T regulatory response FoxP3, IL-10, and TGFβ and expression of effector cytokines IFNγ and IL-17 A analysed by quantitative PCR are shown. Data presented are derived from five to six independent experiments with four to eight mice per group. Kruskal-Wallis test followed by Dunn’s test for multiple comparisons. *p ≤ 0.05, **p ≤ 0.01, ***p ≤ 0.001. Horizontal lines represent medians.

When analysing adaptive immune responses of mice infected for one month with *H*. *pylori* wt or ΔgGT, we observed that gGT-positive *H*. *pylori* induced significantly stronger infiltration of CD3^+^ T cells into the stomach mucosa compared to colonizing *H*. *pylori* devoid of gGT activity (Fig. 3a), even though T cell infiltration was induced by *H*. *pylori* ΔgGT as well. Thus, gGT contributed to enhance influx of T cells in the infected stomach. CD3^+^ T cells were mainly localized at the cardia and in antrum, whereas they were rarely found in the corpus. After six months, the effect of the presence of gGT activity on the recruitment of T cells was even more pronounced, since T cells accumulated over time during infection with *H*. *pylori* wt (Supplementary Fig. S2a,c).

Recruitment of T cells is determined by chemokines and the corresponding receptors guiding activated T cells to the sites of infection. RANTES (CCL5), a chemokine attracting T cells, is also induced in gastric epithelial cells during *H*. *pylori* infection^32^. Expression of RANTES was induced 9.8-fold by colonization with *H*. *pylori* wt, while during colonization with *H*. *pylori* ΔgGT, its levels were comparable to those observed in uninfected animals (Fig. 3b). Lack of RANTES expression may, in part, account for reduced infiltration of T cells observed in absence of gGT.

As we observed that T cells were differentially recruited in absence of gGT activity, their phenotype was further analysed by flow cytometry of stomach homogenates. The proportion of CD3^+^ T cells within the CD45^+^ leukocyte population was increased upon colonization with *H*. *pylori* wt as well as ΔgGT (Fig. 3c). However, influx of CD3^+^ T cells was significantly enhanced in mice colonized with *H*. *pylori* wt compared to ΔgGT (Fig. 3c), confirming the results observed on tissue sections. Furthermore, we observed that CD4^+^ T cells were recruited to the stomach during infection with *H*. *pylori*. While the proportion of CD4^+^ T cells from the CD45^+^ leukocyte population was similar in mice colonized by *H*. *pylori* wt or ΔgGT, infiltration of non-CD4^+^ T cells - most likely CD8^+^ T cells - were more strongly induced by gGT-positive *H*. *pylori* (Fig. 3c).

Even though the proportion of CD4^+^ T cells with respect to the CD45^+^ population was similar in *H*. *pylori* wt and ΔgGT infected mice, the subtypes of the T helper cell population can differ. Since it had been suggested that *H*. *pylori* gGT activity promotes the establishment of a regulatory T cell (Treg) response^24^, FoxP3 expression and proportion of FoxP3-expressing CD4^+^ T cells in gastric tissue of infected mice were analysed. In mice colonized with *H*. *pylori*, infiltration of Foxp3 cells was slightly (not significantly) induced, independently of the gGT status. Interestingly, the proportion of FoxP3-expressing cells within the CD4^+^ T cell population correlated with CFU (Spearman’s r = 0.7; p = 0.0204) (Fig. 3d). This indicates that the abundance of other bacterial factors (not gGT) contributes to Treg induction. In accordance with this, FoxP3 mRNA expression was significantly induced also in mice colonized with *H*. *pylori* ΔgGT (Fig. 3e). In addition to FoxP3, we studied expression of cytokines that cooperate in Treg responses, specifically IL-10 and TGFβ. Both cytokines were slightly, yet significantly, induced, also in mice colonized with gGT-negative *H*. *pylori* (Fig. 3e). Thus, the Treg response was induced to a similar extent in mice colonized with *H*. *pylori* wt or ΔgGT.

We next analysed IFNγ and IL-17 levels as surrogates for Th1 and Th17 responses. Notably, *H*. *pylori* wt and ΔgGT-infected mice showed a distinct expression pattern of proinflammatory Th1 and Th17 effector cytokines. Infection with *H*. *pylori* wt caused a 13.1-fold and 2.2-fold increase in IFNγ and IL-17 A expression in the gastric mucosa, respectively (Fig. 3f). In the absence of gGT, IL-17 A expression but not IFNγ expression was induced by *H*. *pylori*. Thus, IFNγ but not IL-17 A expression was dependent on the presence of gGT. Taken together, these results demonstrate that *H*. *pylori* ΔgGT is not defective in eliciting a Treg response and it induces a similar CD4^+^ Th cell response compared to wt bacteria. However, IFNγ-expression is strongly dependent on gGT activity, suggesting that CD4^+^ T cells might not be the major source of IFNγ during infection.

### *H*. *pylori* gGT induces strong gastric infiltration of CD8^+^ T cells

**Figure 4:**
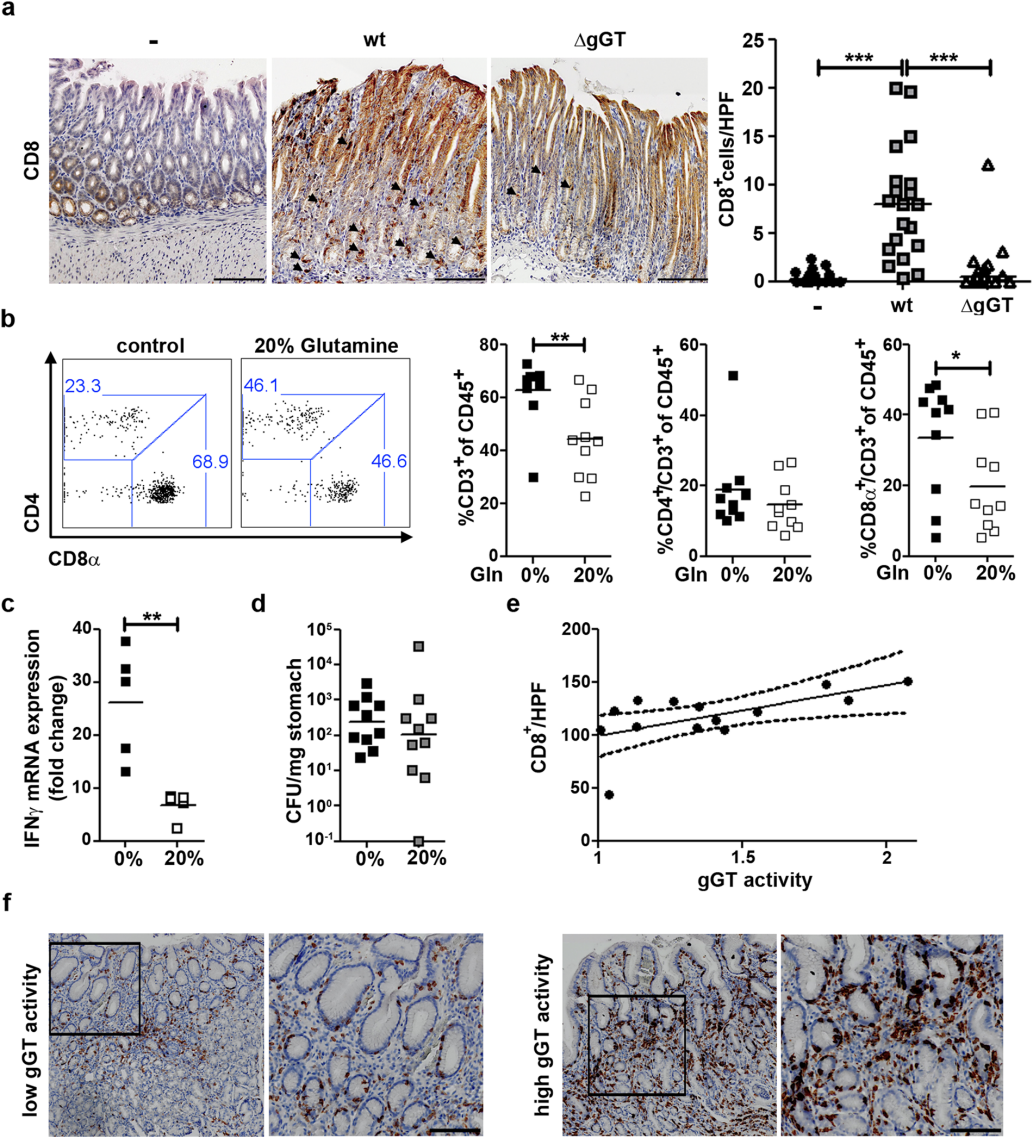
Presence of *H*. *pylori* gGT induces CD8^+^ T cell infiltration in a glutamine-dependent manner. (**a**) Gastric tissue samples from mice infected with *H*. *pylori* wt or ΔgGT for one month were analysed for CD8^+^ cells by immunohistochemistry. Quantification of CD8^+^ cells per high power field (HPF) from three independent experiments was performed. (**b**) Infiltration of CD4^+^ and CD8α^+^ T cells in the stomach analysed by flow cytometry after one month of infection with *H*. *pylori* wt. One group of mice received a glutamine-enriched (20% [w/w]) diet compared to a group of mice that were fed a control diet (0%). Data are derived from two experiments with five mice per group. (**c**) IFNγ mRNA levels and (**d**) colony forming units (CFU) per mg of tissue in stomach samples of mice following a control (0%) or a glutamine-enriched (20%) diet. (**e**) Spearman’s correlation between gGT activity of freshly isolated *H*. *pylori* strains *in vitro* and CD8^+^ cells infiltrating corresponding human stomach samples. r = 0.599; p = 0.0236. (**f**) Representative CD8 staining of human gastric biopsies. Scale bar 100 µm. Mann-Whitney U test ([A]-[C]). *p ≤ 0.05, **p ≤ 0.01. Horizontal bars indicate medians.

As the differential gastric expression of IFNγ between mice colonized by *H*. *pylori* wt bacteria and mice colonized by *H*. *pylori* ΔgGT could not be attributed to differences in infiltrating CD4^+^ T cells, we next studied the influx of CD8^+^ T cells. CD8^+^ T cells produce high levels of IFNγ and could account for IFNγ production during *H*. *pylori* infection. Indeed, we observed higher infiltration of CD8^+^ cells in the stomach of mice infected with *H*. *pylori* wt compared to animals infected with gGT-deficient bacteria (Fig. 4a and Supplementary Fig. S2a). This indicates that gGT favours recruitment of CD8^+^ cells to the stomach.

We and others have shown that glutaminase activity of gGT may affect the viability of host cells. In fact, gGT-mediated glutamine deprivation in the gastric mucosa may compromise the epithelial barrier, induce proinflammatory NFκB signalling, and subsequently cause alterations in the migration pattern of immune cells. To compensate for glutamine deprivation induced by gGT activity, mice were fed a chow enriched with 20% glutamine or an isonitrous control diet during infection. Remarkably, supplementation of glutamine reduced infiltration of T cells and in particular of CD8^+^ T cells into the stomach, while CD4^+^ T cells were unaffected (Fig. 4b). Reduced CD8^+^ T cell infiltration correlated with reduced levels of IFNγ (Fig. 4c) suggesting that CD8^+^ cells are a major source of IFNγ during *H*. *pylori* infection. These effects could not be attributed to differences in bacterial burden since mice fed with the 20% glutamine enriched food presented similar colonization levels (Fig. 4d).

In order to study whether gGT activity was also linked to differential recruitment of T cells into the stomach in humans, we first analysed gGT activity of several clinical isolates. All clinical isolates tested showed gGT activity, but at different levels (Supplementary Fig. S3a). In parallel, we stained the corresponding gastric biopsies, from which the bacteria had been isolated, for CD3^+^, CD4^+^, and CD8^+^ cells. Although no differences in CD3^+^ cells infiltrating the gastric tissue were detected between samples showing high or low gGT activity (Supplementary Fig. S3b), we observed a significant correlation between CD8^+^ cells infiltrating the gastric tissue and gGT activity of the corresponding clinical isolates (Spearman’s r = 0.599; p = 0.0236) (Fig. 4e,f). Thus, clinical isolates showing higher gGT activity induced stronger recruitment of CD8^+^ cells into the gastric mucosa. In contrast, no correlation between gGT activity and infiltration of CD4^+^ cells was detected (Supplementary Fig. S3c). Taken together, this indicates that gGT activity favours the recruitment of CD8^+^ cells to the stomach of *H*. *pylori* infected individuals, as was observed in mice.

## Discussion

*H*. *pylori* is a highly effective pathogen colonizing the hostile niche of the stomach in every second human worldwide. For that, the bacteria employ a panel of sophisticated virulence traits not only to establish an acute infection but also to achieve persistent colonization. While the importance of certain bacterial adhesins is well appreciated in this process, much less is known about metabolic bacterial factors. In this study, we investigated *H*. *pylori* gGT, an enzyme with highly conserved function among *H*. *pylori* strains. All clinical isolates seem to harbour and express active gGT, suggesting an important role for this bacterial factor during infection. While studies of *H*. *pylori* in humans are generally limited to subjects with established infection, murine infection models offer the great advantage that infection in presence and absence of gGT can be studied. Previous studies in mice were not conclusive regarding the question of whether *H*. *pylori* gGT is mainly required for initial colonization or for the establishment of a persistent infection^24–26^. Differences among these studies regarding colonization capacity of *H*. *pylori* ΔgGT are likely due to different strains used and the variability in colonization rate from independent inoculations.

By analysing bacterial load at day three and manipulating the presence of enzymatic activity, we found evidence that gGT mainly determines colonization capacity during initial colonization. The study of colonization levels for up to six months revealed that *H*. *pylori* ΔgGT was capable to colonize persistently in mice once it had established an infection. While further metabolomic analyses are necessary to explore what discriminates those gGT-deficient bacteria that were able to colonize from those that failed to establish an infection, our results indicate that *H*. *pylori* gGT activity provides an advantage to the bacterium favouring initial colonization. Several functional roles of *H*. *pylori* gGT by which the enzyme may support the bacteria have been suggested. gGT provides nitrogen and carbon sources for bacterial metabolism and contributes to acid resistance through release of ammonium^20,21,33^. Considering that *H*. *pylori* ΔgGT is not growth-deficient *in vitro*, the stomach environment seems to pose a specific hurdle for colonization that is overcome with aid of gGT-activity. *H*. *pylori* gGT may modify the availability of amino acids or produce neutralizing ammonia. In humans, *H*. *pylori* seems to depend even more on gGT activity as no gGT-deficient human isolate has been described. This indicates that a stronger selective pressure, such as higher acidity or fewer alternative nutrients, is encountered in the stomach of humans compared to mice. The notion that *H*. *pylori* gGT was important to establish an infection in the first place is supported by the absence of signs for an innate or adaptive immune defence at three days post infection. In addition, a similar initial hurdle was also observed in Rag^−/−^ mice, where adaptive immune cells are absent.

Direct effects of *H*. *pylori* gGT on gastric epithelial and immune cells had been proposed from previous studies. On the one hand, toxic effects on gastric epithelial cells due to oxidative stress and ammonia production promoting proinflammatory pathways have been observed^19,34,35^. On the other hand, *H*. *pylori* gGT has been demonstrated to drive dendritic cells towards a tolerogenic phenotype^23,24^ and to suppress effector T cell activity in a glutamine-dependent manner^22^. The contributions of these effects during infection are still elusive.

Given the finding that mice were colonized with *H*. *pylori* wt or ΔgGT at a comparable level at one and six months in this study, changes in the immune response could be attributed to gGT activity (rather than colonization density). At a similar bacterial burden, other bacterial factors known to influence the immune response, e.g. VacA and CagA, are assumed to be present at similar levels. In humans, IL-8, but not ENA-78/CXCL5 expression, has been shown to be associated with CagA status^28^. Here, we demonstrate that the induction of the murine CXCL5 homologue, LIX, is strongly dependent on the presence of gGT. CXCL5 is involved in the regulation of CXCR2-dependent neutrophil trafficking and has been associated with the pathology of inflammatory bowel disease in human and DSS-induced colitis in mice^36,37^. Thus, we hypothesize that induction of CXCL5 may be one mechanism by which gGT contributes to gastric inflammation.

Surprisingly, T helper cell responses were not altered in mice colonized with the gGT-deficient *H*. *pylori* strain, since CD4^+^ T cell infiltration, IL-17 A expression, and Treg markers were comparable to mice infected with the wt strain. However, IFNγ expression and infiltration of CD3^+^ T cells (to a lesser extend) were induced stronger in mice colonized with gGT-proficient bacteria. IFNγ plays a major role in *H*. *pylori*-associated gastritis, as previous studies with IFNγ-deficient mice have shown^10,11^. Since infiltration of CD4^+^ T cells was not changed, we assume that IFNγ might originate from CD8^+^ T cells. Interestingly, Ruiz *et al*. reported a dominance of CD8^+^ T cells over CD4^+^ T cells during *H*. *pylori* infection and that a higher proportion of CD8^+^ T cells compared to CD4^+^T cells isolated from the stomach of *H*. *pylori* PMSS1-infected mice produced IFNγ upon CD3/CD28 and PMA/Ionomycin stimulation^38^. This concurs with results presented in this study, as we found a greater proportion of CD8^+^ T cells compared to CD4^+^T cells in the stomach of *H*. *pylori*-infected mice, which was accompanied by strong expression of IFNγ. Notably, infiltration of CD8^+^ T cells into the gastric mucosa of infected mice as well as IFNγ expression was highly dependent on the presence of gGT. This was corroborated by our findings in human tissue revealing that infiltration of CD8^+^ T cells correlated with higher gGT activity. Therefore, we sought to determine how gGT activity may influence CD8^+^ T cell levels in the stomach. Interestingly, gGT-induced effects on gastric epithelial cells have been attributed to glutamine deprivation and toxic ammonia^19,35^. To counteract gGT-induced glutamine deprivation in the gastric mucosa, we supplemented glutamine during infection with *H*. *pylori*. It has been reported before that glutamine administration protects rodents against *H*. *pylori*-induced pathology^39,40^, even though it had not been linked to gGT activity. Remarkably, gGT-induced effects were reverted by administration of glutamine, as infiltration of CD8^+^T cells was strongly reduced, while bacterial load was unaffected. This suggests that gGT-dependent glutamine deprivation plays a pivotal role in promoting *H*. *pylori*-induced mucosal inflammation and differential recruitment of T cells into the stomach. Glutamine deprivation was shown to stimulate mTOR-JNK-dependent chemokine secretion^41^, to trigger an endoplasmic reticulum (ER) stress response, and to induce NFκB activation - all culminating in the induction of the proinflammatory chemokine IL-8^42^. Notably, a subset of CD8^+^ T cells bearing the CXC chemokine receptor 1 (CXCR1) was described. These cells responded to IL-8 and expressed perforin, granzyme B, and IFNγ, and had high cytotoxic potential^43^.

In human gastric tissue, CD4^+^ as well as CD8^+^ T cells are increased upon *H*. *pylori* infection^5,8,44^. Moreover, CD8^+^ T cells isolated from *H*. *pylori*-infected patients produced more IFNγ than CD4^+^ T cells on a per cell basis upon antigen restimulation^45^. In experimental *H*. *pylori* infection of mice, CD8^+^ as well as CD4^+^ T cells contribute to control of colonization since absence of either T cell subtype, due to MHC class I or II deficiency, led to a similar increase in bacterial burden compared to wt mice^46^. However, most research focused on CD4^+^ T cells and neglected possible clinically relevant roles of CD8^+^ T cells. Our results suggest a significant role for CD8^+^ T cells in the course of *H*. *pylori* infection, which has been overlooked so far and merits further characterization.

Taken together, our results provide evidence that the enzymatic activity of gGT is not only important for initial colonization by the bacterium, but that it profoundly changes the stomach milieu instructing differential recruitment of immune cells into the gastric mucosa, which can have a major impact in the final outcome of the infection.

## Methods

### Bacterial infection of C57Bl/6 mice with *H*. *pylori*

Six- to eight-week old female C57Bl/6 wild-type (wt) mice were obtained from Harlan. C57Bl/6 Rag^−/−^ mice were derived from in-house breeding. Mice were housed under specific pathogen-free conditions and maintained under microisolator cages with chow and water ad libitum. Experiments were conducted in compliance with European guidelines for the care and use of laboratory animals and were approved by the local authorities (Regierung von Oberbayeren [AZ 55.2.1-54-2532-147-12]). Prior to infection, mice were fasted for four to six hours. *H*. *pylori* bacteria (2 × 10^9^ CFU) were administered to mice once by oral gavage in 200 µl Brucella broth containing 10% FCS. The *H*. *pylori* PMSS1 strain and its isogenic mutant hp1118::kan (ΔgGT) was used^47^. Mice were sacrificed after three days, one month, or six months post infection, and gastric tissue was collected for histologic examination, quantitative bacterial culture, mRNA expression analysis, and flow cytometry.

### Experimental design, administration of recombinant gGT

Mice inoculated with *H*. *pylori* ΔgGT, as described above, were administered recombinant *H*. *pylori* gGT (rgGT)^22^. Groups of four mice orogastrically received 50 µg or 250 µg rgGT (150 µl in PBS) at the time of infection and on the two consecutive days (3x). One group that had received the high dose (250 µg) was only treated once. A control group was only given bacteria. Colonization level was analysed three days after infection.

### Experimental design, supplementation of glutamine

For supplementation of glutamine, mice infected with *H*. *pylori* wt or ΔgGT received research diets enriched with 20% glutamine or an isonitrous control diet (Ssniff Research Diet). Chow was provided ad libitum during the infection period. Mice were infected with *H*. *pylori* wt or ΔgGT for one month. Control groups received the respective diets but were not given bacteria.

### Assessment of bacterial colonization

For colonization levels, longitudinal gastric tissue sections were weighted and homogenised in Brucella broth containing 10% FCS. Then, number of colony forming units (CFU) was determined by plating serial dilutions on WC-dent agar plates supplemented with bacitracin (200 µg/ml), nalidixic acid (10 µg/ml), and polymyxin B (3 µg/ml).

### Histologic examination of gastric tissue

For histology of murine stomachs, a longitudinal strip was cut from the oesophagus to the duodenum, along the lesser curvature, and back to the oesophagus along the longer curvature. Gastric tissue was fixed with formalin and embedded in paraffin. Human gastric tissue samples were obtained from the Institut für Pathologie, Klinikum München-Bogenhausen, after approval of the ethical committee of the Klinikum rechts der Isar. Informed consent was obtained from all subjects undergoing gastric endoscopy. All experiments were performed in accordance with relevant guidelines and regulations.

For histologic evaluation haematoxylin and eosin (HE), chloroacetate esterase staining (CAE) and immunohistochemistry were performed. Pathology score was determined according to the updated Sydney score system^48^.

For immunohistochemistry, sections were dewaxed in xylene and gradually rehydrated 50-100% ethanol. Heat-induced antigen retrieval was performed in 0.01 M sodium citrate pH 6 and slides were blocked in 5% goat serum for 1 h at room temperature. A primary monoclonal CD3 antibody was purchased from Neomarkers LabVision. A CD8 antibody from Thermo Scientific was used to stain human samples while a CD8 antibody from Dianova was used on murine samples. Gastric sections were incubated with primary antibodies overnight at 4 °C following manufacturer’s instructions. HRP-conjugated secondary antibody was applied for 1 h and samples were developed using SignalStain DAB substrate (Cell Signaling). Murine sections were stained on an automated staining machine (Bondmax Rxm) with 15 min incubation of the primary antibody, a secondary rabbit-anti-rat antibody (Vector Laboratories) and the Polymer Refine Detections system (Leica). Sections were counterstained with haematoxylin. Automated image acquisition was performed using the Virtual Slide Scanning System VS120 (Olympus). Five high power fields (20x magnification) were randomly scored for each sample.

### Flow cytometry

Gastric samples were placed in RPMI (Gibco) containing 10% FCS and processed using DNase (1 mg/ml, Roche Diagnostics) and Collagenase IV (200 µg/ml, Sigma-Aldrich) digestion for 30 min at 37 °C under rapid shaking to isolate immune cells. Cells were treated with ethidium monoazide for live/dead discrimination. Then, surface antigens were stained using following fluorochrome-conjugated antibodies (eBioscience): CD45-APC, CD3-PECy7, CD4-eF450, and CD8α-APC-Cy7. For flow-cytometric detection of FoxP3, cells were stained for cell surface markers, fixed, permeabilized, and stained with a PE-conjugated FoxP3 antibody (eBioscience). After incubation, cells were fixed with 0.5% paraformaldehyde. Data were acquired by a CyAN ADP 9 color analyzer (Beckman Coulter) and analysed using Flow Jo software (Tree Star).

### Real-time quantitative PCR

Total RNA was isolated from homogenized gastric tissue using the RNeasy Mini Kit (Qiagen) according to the manufacturer’s instructions including on-column DNase digestion. RNA integrity was confirmed by agarose gel electrophoresis. RNA was converted to cDNA using a combination of random hexamer primers and M-MLV reverse transcriptase (Promega). Subsequently, cDNAs were amplified using a CFX384 qPCR cycler (Bio-Rad). The sequences of the gene-specific primers used were as follows: GAPDH sense primer, 5′-GCCTTCTCCATGGTGGTGAA-3′; GAPDH antisense primer, 5′-GCACAGTCAAGGCCGAGAAT-3′; IFNγ sense primer, 5′-TCAAGTGGCATAGATGTGGAAGAA-3′; IFNγ antisense primer, 5′-TGGCTCTGCAGGATTTTCATG-3′; IL-17 A sense primer, 5′-GCTCCAGAAGGCCCTCAGA-3′; IL-17 A antisense primer, 5′-AGCTTTCCCTCCGCATTGA-3′; TNFα sense primer, 5′-CGATGGGTTGTACCTTGTC-3′; TNFα antisense primer, 5′-CGGACTCCGCAAAGTCTAAG-3′; LIX sense primer, 5′-GGTCCACAGTGCCCTACG-3′; LIX antisense primer, 5′-GCGAGTGCATTCCGCTTA-3′; MIP-2 sense primer, 5′-AGTGAACTGCGCTGTCAATGC-3′; MIP-2 antisense primer, 5′-AGGCAAACTTTTTGACCGCC-3′; IL-1β sense primer, 5′-CAACCAACAAGTGATATTCTCCATG-3′; IL-1β antisense primer, 5′-GATCCACACTCTCCAGCTGCA-3′; FoxP3 sense primer, 5′-AGGAGCCGCAAGCTAAAAGC-3′; FoxP3 antisense primer, 5′-TGCCTTCGTGCCCACTGT-3′; TGFβ sense primer, 5′-ATCCTGTCCAAACTAAGGCTCG-3′; TGFβ antisense primer, 5′-ACCTCTTTAGCATAGTAGTCCGC-3′; IL-10 sense primer, 5′-CTAGAGCTGCGGACTGCCTTC-3′; IL-10 antisense primer, 5′-CCTGCTCCACTGCCTTGCTCTTAT-3′; RANTES sense primer, 5′-CACCACTCCCTGCTGCTT-3′; RANTES antisense primer, 5′-ACACTTGGCGGTTCCTTC-3′. GAPDH was used as an endogenous control to normalise the target gene expression. Then, fold change was calculated using the 2^−ΔΔCT^ method.

### gGT activity assay

*H*. *pylori* gGT activity was measured using a colorimetric assay previously described^27^. 2 × 10^8^ bacteria were resuspended in 1 ml of PBS and incubated at 37 °C (5% CO^2^, 110 rpm) for 2 h. After centrifugation (13.000 rpm, 10 min, RT), 50 µl of supernatant per well were applied to a 96-well plate. Each well was filled up to a total volume of 200 µl per well with reaction buffer consisting of 5 mM L-gamma-glutamyl-p-nitroanilide (L-gGpNA) as substrate and 100 mM glycylglycine (Gly-Gly) as acceptor in 0.1 M Tris buffer, pH 8. Experiments were conducted in duplicates and performed three independent times. Absorption was monitored at 405 nm using a Mithras LB 940 Multimode Microplate Reader (Berthold Technologies) after 1 h of incubation at 37 °C.

### Statistical analysis

Data are presented as median and nonparametric tests were used to determine statistical significance using Prism 5 software (GraphPad). Data from two experimental groups were compared by the Mann-Whitney U test. Data from more than two groups were analysed using the Kruskal-Wallis test followed by Dunn’s multiple post hoc test for multiple comparisons. Chi-Squared test was used to compare the number of infected and uninfected mice. Correlations were established using Spearman’s rank correlation coefficient. Statistical significance of differences was established when p ≤ 0.05.

## Electronic supplementary material

Supplementary Information
