# Supplementary material for: Helicobacter pylori γ-glutamyl transferase contributes to colonization and differential recruitment of T cells during persistence
Source: Sci Rep. 2017 Oct 20;7:13636. doi: 10.1038/s41598-017-14028-1 (PMC5651840; doi:10.1038/s41598-017-14028-1)
Supplement: Supplementary file 1 — Supplementary Information [file 41598_2017_14028_MOESM1_ESM.pdf]

Helicobacter pylori γ-glutamyl transferase contributes to colonization and differential recruitment of T cells during persistence.

Stefanie Wüstner[1], Florian Anderl[1], Andreas Wanisch[1], Corinna Sachs[1], Katja Steiger[2], Andreas Nerlich[3], Michael Vieth[4], Raquel Mejías-Luque[1,5,¶,*] and Markus Gerhard[1,5,¶,*]

[1]Institut für medizinische Mikrobiologie, Immunologie und Hygiene, Technische Universität München, 81675 Munich, Germany.

[2]Institute of Pathology, Technische Universität München, 81675 Munich, Germany.

[3]Institut für Pathologie, Klinikum München-Bogenhausen, 81925 Munich, Germany.

[4]Institut für Pathologie, Klinikum Bayreuth, 95445 Bayreuth, Germany.

[5]German Centre for Infection Research (DZIF), partner site Munich, Munich, Germany.

[¶]These authors contributed equally to this work.

[*]**Correspondence:**

raquel.mejias-luque@tum.de / markus.gerhard@tum.de

Supplementary Figure 1. Confirmation of *H. pylori* ΔgGT mutant and assessment of pathology at day three. (**a**) Colonization level of mice inoculated with *H. pylori* PMSS1 ΔgGT was assessed by plating serial dilutions on plates without and with 50 µg/ml kanamycin. Number of colony forming units (CFU) from each sample plated on either plate did not differ according to Wilcoxon signed rank test. (**b**) Inflammatory score in stomach of mice at three days post infection. (**c**) Chloroacetate esterase (CAE) staining and $CD3^+$ cells detected by immunohistochemistry in gastric tissue samples after three days of infection. (**d**) C57Bl/6 mice were inoculated with *H. pylori* wt, ΔgGT, or a combination of both. Bacterial load was analysed after one month. Data are derived from three independent inoculations (four to eight mice per group). (**e**) PCR of the gGT locus that encompasses the kanamycin resistance cassette. (**f**) gGT activity of *H. pylori* PMSS1 parental strains and reisolates. (**g**) C57Bl/6 mice were infected with *H. pylori* wt or ΔgGT parental strain or *H. pylori* ΔgGT that had been isolated from mice that had been colonized with *H. pylori* ΔgGT before for ten days (d10) or one month at high and low levels. Mice were analysed at one month post infection.

Supplementary Figure 2. Long-term effects of gGT deficiency on gastric immunopathology. Mice were infected with *H. pylori* wt or ΔgGT for six months. (**a**) Representative hematoxylin-eosin (HE), chloroacetate esterase (CAE) and CD3 and CD8 stainings of gastric tissue samples. (**b**) Number of neutrophils (CAE^+ cells) per high power field (HPF) in the stomach of control and infected mice. (**c**) $CD3^+$ T cells per HPF observed in the stomach of mice.

Kruskal-Wallis test followed by Dunn's test for multiple comparisons. $^*p \leq 0.05$, $^{**}p \leq 0.01$. Horizontal bars indicate medians.

Supplementary Figure 3. Correlation of gGT activity and gastric infiltration of T cells. (**a**) gGT activity of *H. pylori* strains isolated from human gastric biopsies (C1-14) and mouse adapted control strains (G27 and PMSS1). (**b**) Spearman's correlation between gGT activity of *H. pylori* strains isolated from human gastric biopsies and $CD3^+$ cells infiltrating corresponding tissue samples. p=0.8873. (**c**) Spearman's correlation between gGT activity of

H. pylori strains isolated from human gastric biopsies and $CD4^+$ cells infiltrating corresponding tissue samples. p=0.2140.

Supplementrary Figure S1

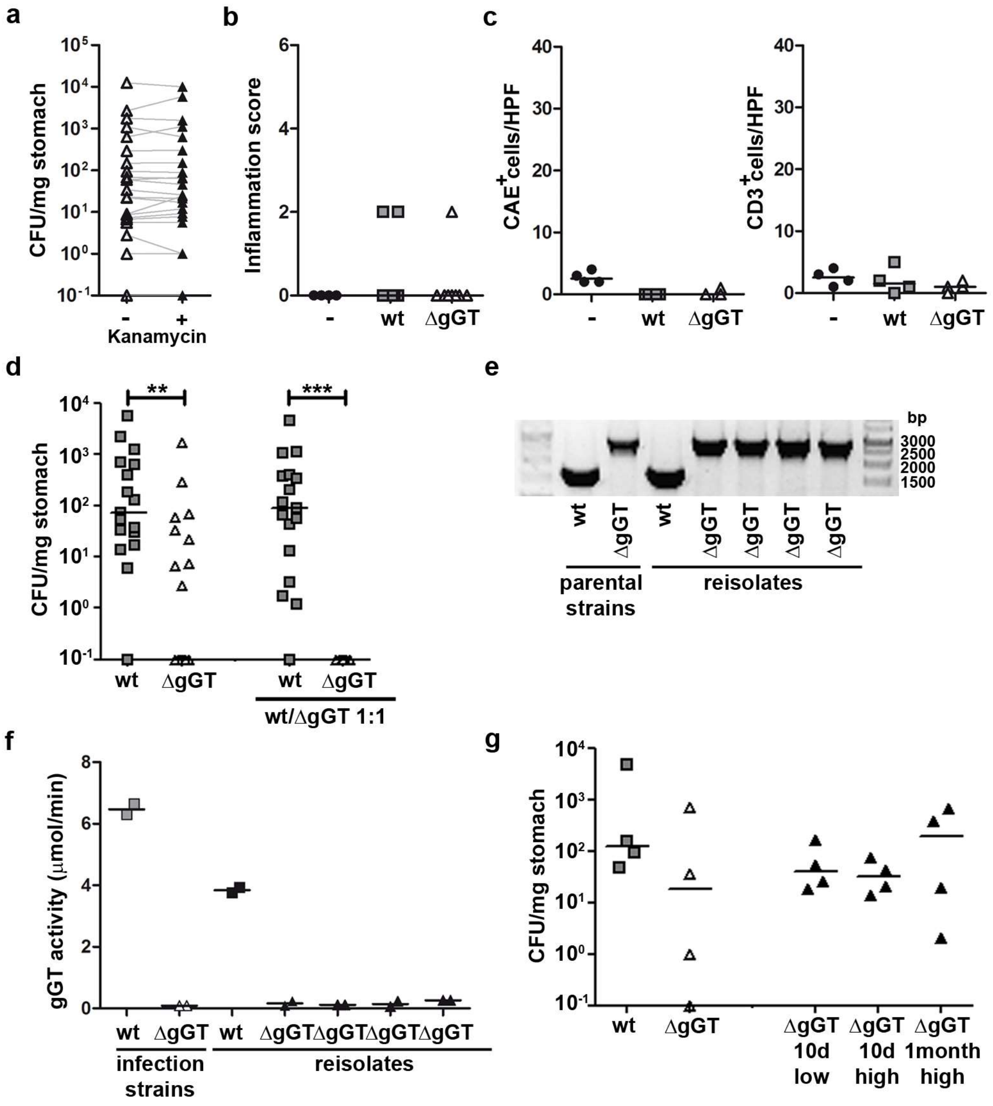

Supplementary Figure S2

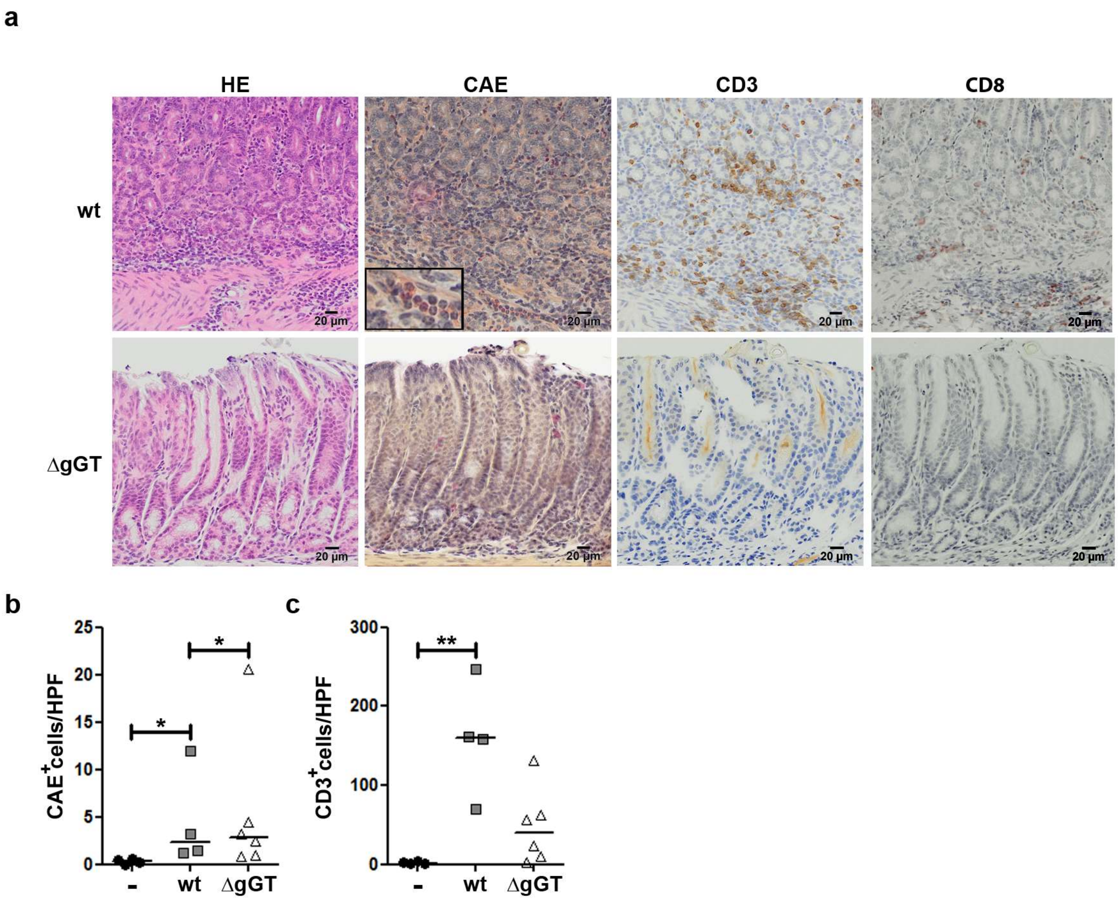

Supplementary Figure S3

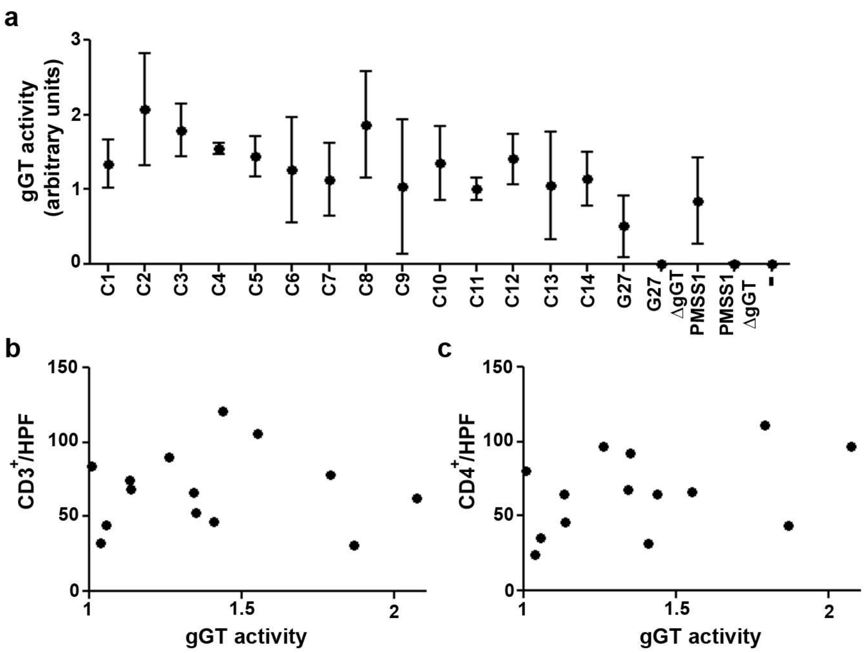

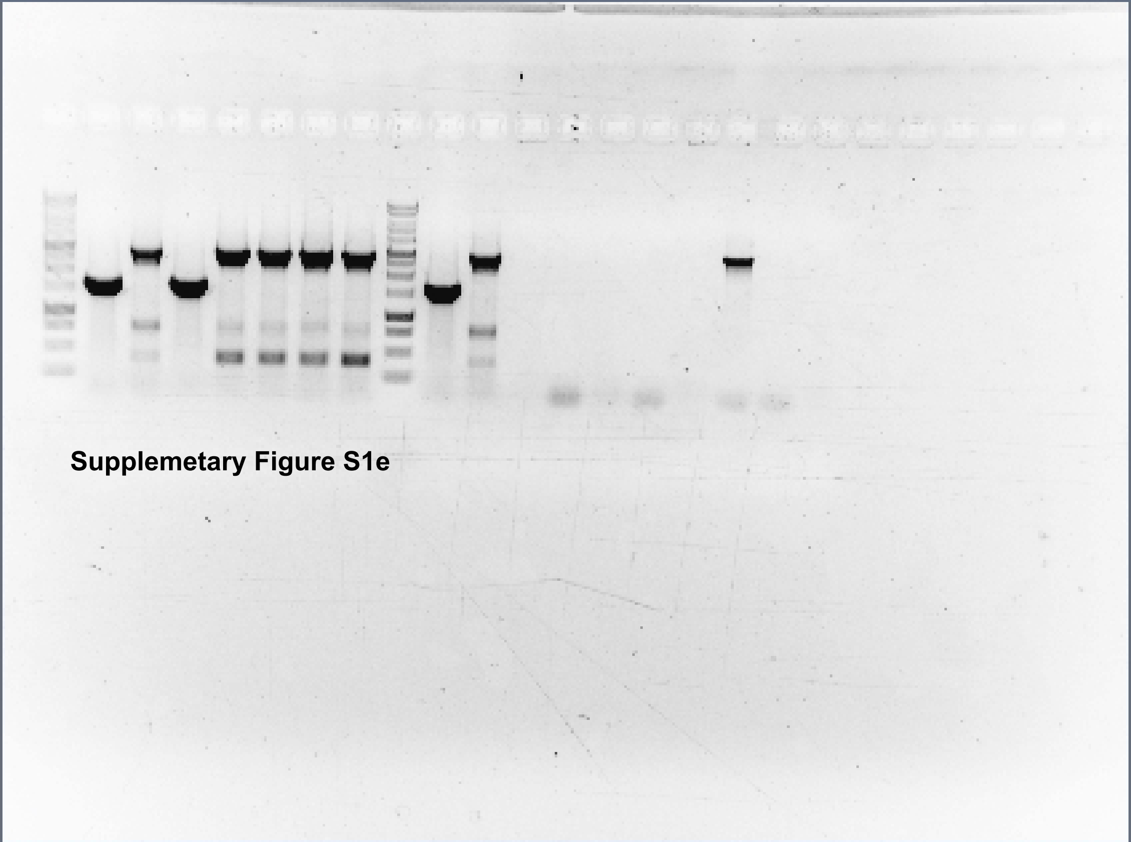

Supplemetary Figure S1e
